# HD-AIP: a heterogeneous dual-stream alignment-free framework for anti-inflammatory peptide prediction based on language models and CT-Net

**DOI:** 10.1093/bioinformatics/btag697

**Published:** 2026-09-21

**Authors:** Jiangli Li, Quan Zou, Yansu Wang, Yifeng Bai, Hao Zhou, Mengting Niu

**Affiliations:** School of Computer Science and Engineering (School of Cyber Security), University of Electronic Science and Technology of China, Chengdu, Sichuan 611731, China; Institute of Fundamental and Frontier Sciences, University of Electronic Science and Technology of China, Chengdu, Sichuan 610054, China; Yangtze Delta Region Institute (Quzhou), University of Electronic Science and Technology of China, Quzhou, Zhejiang 324003, China; Institute of Fundamental and Frontier Sciences, University of Electronic Science and Technology of China, Chengdu, Sichuan 610054, China; Yangtze Delta Region Institute (Quzhou), University of Electronic Science and Technology of China, Quzhou, Zhejiang 324003, China; Department of Oncology Sichuan Provincial People’s Hospital, University of Electronic Science and Technology of China, Chengdu, Sichuan 610072, China; Department of Stomotology Sichuan Provincial People’s Hospital, University of Electronic Science and Technology of China, Chengdu, Sichuan 610072, China; School of Electronic and Communication Engineering, Shenzhen Polytechnic University, Shenzhen, Guangdong 518055, China

## Abstract

**Motivation:**

Anti-inflammatory peptides (AIPs) show therapeutic potential for treating chronic and autoimmune diseases. Computational screening of these peptides remains challenging because their typically short sequences limit traditional feature extraction effectiveness, while homology-based methods incur high computational costs.

**Results:**

This study proposes HD-AIP, an alignment-free heterogeneous dual-stream prediction architecture. This framework extracts peptide features in parallel from both macroscopic and microscopic perspectives. Macroscopically, HD-AIP integrates global semantic features extracted by two large protein language models, ProtT5 and ESM-2 3B, with sequence-level physicochemical properties, followed by feature selection and LightGBM classification. Microscopically, an asymmetric parallel network named CT-Net utilizes BioVec embeddings and residue-level physicochemical features, using a CNN branch to capture local motifs and a Transformer branch to model long-range dependencies. The two streams are adaptively fused via a dynamic soft ensemble strategy. On an independent test set, HD-AIP outperforms baseline models across multiple metrics including accuracy, area under the receiver operating characteristic curve, and the Matthews correlation coefficient. These results indicate that HD-AIP improves AIP prediction performance without sequence alignment. This architecture serves as an effective computational tool for the high-throughput virtual screening and candidate discovery of AIPs.

**Availability and implementation:**

You can find the source code and dataset needed on GitHub (https://github.com/Zerofly0/HD-AIP). The required package files have been listed in the readme.

## 1 Introduction

Inflammation is an immune defense response to harmful stimuli, crucial for maintaining tissue homeostasis. However, excessive or persistent chronic inflammation is a core trigger for various severe pathological conditions, including rheumatoid arthritis, Alzheimer’s disease, and cancer (Nathan and Ding 2010). Although traditional non-steroidal anti-inflammatory drugs and corticosteroids demonstrate significant clinical efficacy, long-term use frequently causes severe side effects and drug resistance (Bjarnason *et al.* 2018). Consequently, anti-inflammatory peptides (AIPs) emerge as a highly promising therapeutic alternative due to their high specificity, strong efficacy, and low toxicity. Nevertheless, identifying these peptides from vast candidate sequences via traditional wet-lab experiments, such as *in vivo* or *in vitro* bioassays, is extremely time-consuming and costly. As high-throughput sequencing technologies accumulate massive amounts of protein sequence data, developing efficient computational prediction tools becomes critical to accelerate peptide drug discovery.

Numerous computational prediction tools have emerged in biomedical research, such as those developed for protein sequence classification (Wang *et al.* 2024) and circRNA-disease association prediction (Niu *et al.* 2025). Beyond predictive tasks, large language model (LLM)-based systems have also been developed to automate dry-lab research workflows (Luo *et al.* 2025). In the field of AIP prediction, the development of computational methods can be broadly divided into three stages. Early research primarily relied on classical machine learning algorithms. AIPpred by Manavalan *et al.* (2018) established an early prediction baseline using random forests combined with dipeptide composition information. Subsequently, researchers attempted to integrate multidimensional physicochemical properties and evolutionary information. PreAIP by Khatun *et al.* (2019) integrated multiple complementary features, while PPTPP by Zhang and Zou (2020) introduced adaptive feature representation learning. Recently, deep learning (DL) technologies significantly enhanced the feature extraction capabilities of models. Guan *et al.* (2023) proposed an ensemble architecture fusing GRU and attention mechanisms, demonstrating the advantages of deep features in processing peptide sequences. Concurrently, to pursue higher prediction accuracy, several leading methods began relying heavily on homology alignment features such as PSSM. For example, DeepAIPs-Pred (Akbar *et al.* 2024) and DeepAIPs-SFLA (Akbar *et al.* 2025) by Akbar *et al.* achieved excellent experimental results by constructing local image features based on evolutionary information.

However, these homology-dependent methods face multiple challenges in practical applications. (i) Obtaining PSSM matrices requires time-consuming iterative alignments against massive protein databases like NR or Swiss-Prot, making computational costs prohibitively high for large-scale virtual screening; (ii) because AIPs typically feature short sequences with highly variable conservation, forced alignment potentially introduces additional noise, leading to high false positive (FP) rates; and (iii) existing models still struggle to balance the macroscopic global semantics and microscopic local motif information of peptide sequences within a single architecture.

To overcome these limitations, this study develops HD-AIP, an alignment-free dual-stream prediction framework. The core innovations are three-fold: (i) alignment-free multi-scale feature construction—without relying on PSSM or homology search, HD-AIP integrates macroscopic global semantics (via ProtT5 and ESM-2 3B) with microscopic local topologies (via BioVec and residue-level physicochemical properties), completely avoiding the computational bottleneck of sequence alignment. (ii) Heterogeneous dual-stream architecture—the framework processes peptides in parallel: a pre-trained protein language model (PLM)-fusion machine learning stream captures global sequence-level properties, while CT-Net—an asymmetric CNN-transformer network—extracts local motifs and long-range dependencies from residue-level features. This design balances global and local information. (iii) Dynamic soft ensemble strategy—instead of stacking or simple averaging, an adaptive weighted fusion dynamically combines the two streams’ prediction probabilities. This avoids overfitting on imbalanced data and improves discriminative robustness on unknown sequences. Ablation studies, comparisons with baseline models, and independent test set validations demonstrate that HD-AIP achieves superior prediction performance and generalization without any sequence alignment. The overall framework is illustrated in Fig. 1.

**Figure 1:**
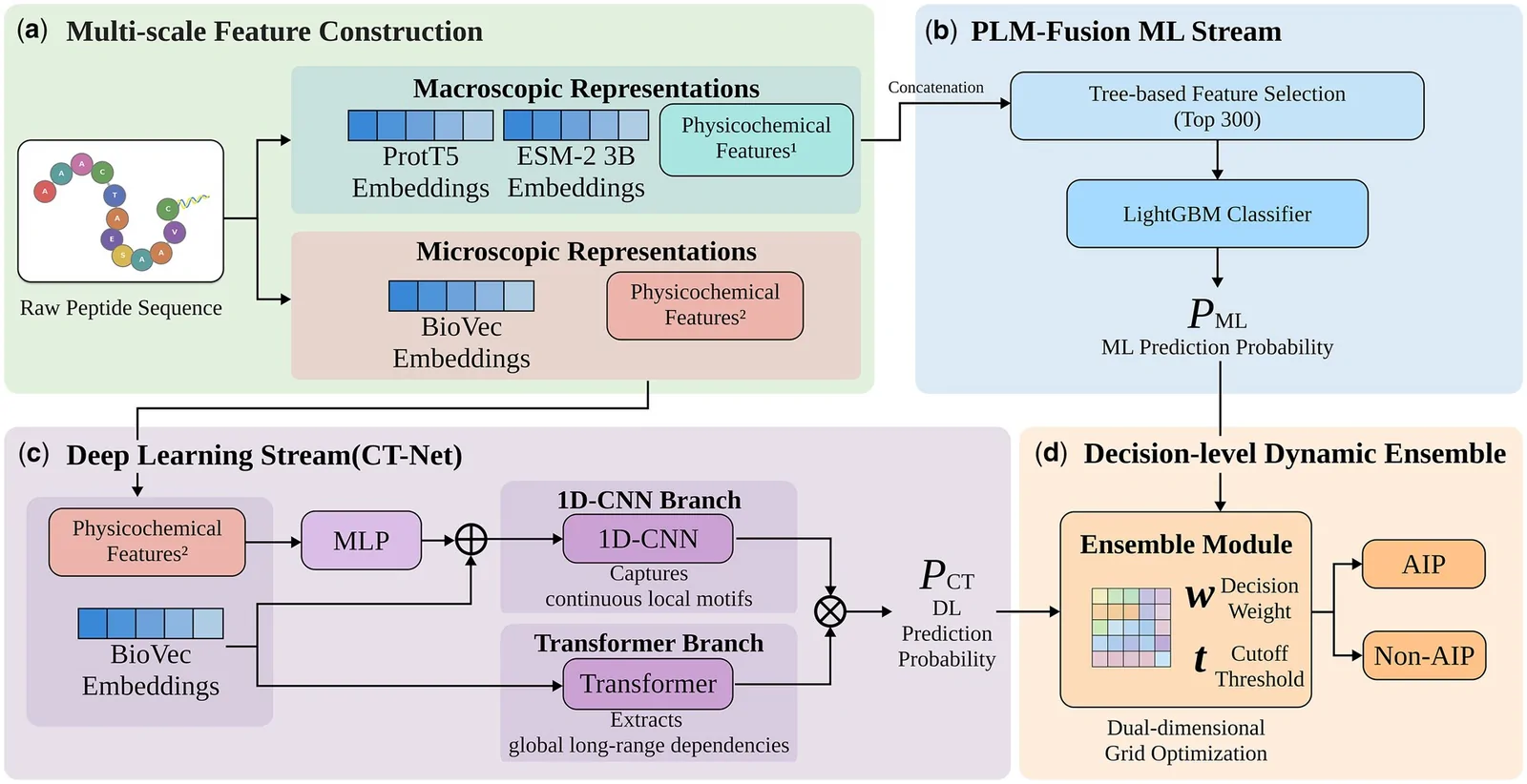
The overall architecture of the HD-AIP prediction framework. (a) Multi-scale feature construction, (b) macroscopic PLM-fusion machine learning stream, (c) microscopic CT-Net DL stream, and (d) dynamic soft ensemble for final prediction.

## 2 Materials and methods

### 2.1 Datasets

To ensure objective evaluation and comparability with existing studies, we utilize datasets from previous studies (Xu *et al.* 2024, Zhao *et al.* 2021). The benchmark dataset was obtained from the Immune Epitope Database (Vita *et al.* 2019), a widely used public resource for epitope data storage and prediction tool development. The construction of this dataset relies on strict biological experimental validation standards. Specifically, the positive samples (AIPs) contain linear peptides and epitopes experimentally validated (via human or mouse T cell assays) to induce cytokines with anti-inflammatory or immunoregulatory functions under specific biological contexts, including IL-10, IL-4, IL-13, IL-22, TGF-β, and IFN-α/β. The negative samples (Non-AIPs) are peptides experimentally confirmed not to stimulate production of these cytokines (Vita *et al.* 2019). This dataset comprises 4194 peptide sequences, all of which serve in the model training and 5-fold cross-validation (FFCV) of the proposed HD-AIP framework. Table 1 presents the class distribution.

**Table 1:** Distribution of the benchmark dataset used in this study.

Dataset	Category	Count
Benchmark dataset	Positive (AIPs)	1678
	Negative (Non-AIPs)	2516
Total	–	4194

To evaluate generalization on unseen sequences, we constructed an independent test set from the test data of the BertAIP study (Xu *et al.* 2024), which originally comprised 1009 peptides. To eliminate the risk of performance overestimation caused by data leakage, we removed any sequence with an exact match to the training set using string matching, yielding a final independent set of 303 peptides. This dataset remains strictly isolated from model training, feature selection, and hyperparameter optimization, serving solely for the final independent testing. Table 2 presents its detailed composition.

**Table 2:** Distribution of the independent dataset used in this study.

Dataset	Category	Count
Independent dataset	Positive (AIPs)	116
	Negative (non-AIPs)	187
Total	–	303

Both the benchmark dataset and the independent test set exhibit moderate class imbalance (Fig. 2a). The benchmark dataset contains 1678 positive and 2516 negative samples, with a positive-to-negative ratio of approximately 1:1.50. Similarly, the independent test set consists of 116 positive and 187 negative samples, presenting a ratio of about 1:1.61. Therefore, we adopted comprehensive metrics including Matthews correlation coefficient (MCC), sensitivity (Sn), specificity (Sp), and the area under the receiver operating characteristic curve (AUC) to avoid evaluation bias. Sequence length distributions demonstrate that the target AIPs are predominantly short, possessing limited contextual information (Fig. 2b). To address the representational sparsity of single-feature methods on short sequences, our framework integrates macroscopic semantic features, explicit physicochemical properties, and microscopic topological features. Finally, distinct amino acid usage preferences between positive and negative (Fig. 2c) samples provide an empirical basis for incorporating composition features and modeling local residue patterns within the architecture.

**Figure 2:**
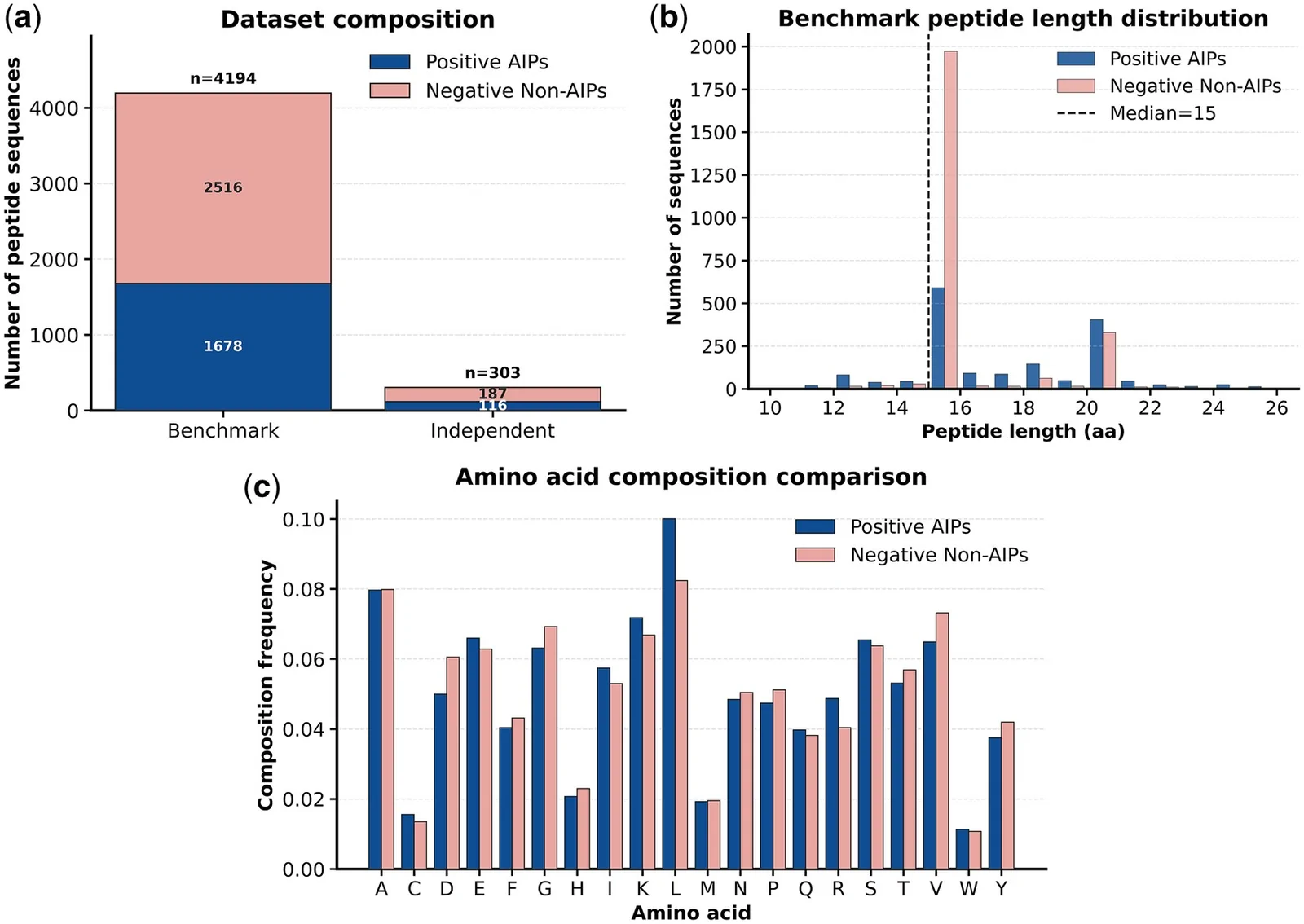
Dataset composition and sequence-level characterization. (a) Class distribution of the benchmark and independent datasets, (b) Peptide length distribution of AIPs and Non-AIPs in the benchmark dataset, and (c) AAC comparison between AIPs and Non-AIPs in the benchmark dataset.

### 2.2 Construction of multi-scale feature space

Prediction performance heavily depends on feature representation. Most state-of-the-art models rely on evolutionary features derived from multiple sequence alignment [e.g. PSSM (Altschul *et al.* 1997)], which requires time-consuming homology searches against large protein databases. This alignment-dependent process incurs high computational costs and creates bottlenecks for high-throughput virtual screening.

To address these issues, this study proposes an alignment-free multi-view feature representation strategy that integrates pre-trained protein language models with complementary explicit physicochemical features.

#### 2.2.1 Pre-trained protein language models

Self-attention-based language models have recently been introduced into the field of protein science. Through self-supervised pre-training on massive unlabeled protein sequences, these models learn local dependencies and global topological constraints among amino acid residues in a data-driven manner. To extract deep implicit semantics from peptide sequences, this study utilizes two mainstream large protein language models.

- ProtT5: we adopt the ProtT5-XL-UniRef50 model based on the T5 architecture (Elnaggar *et al.* 2022), which maps discrete amino acid sequences into a high-dimensional continuous latent semantic space.
- ESM-2: we utilize the ESM-2 model developed by Meta AI (Rives *et al.* 2021), which directly extracts deep evolutionary and structural information from a single input sequence.

An input sequence of length L is processed through both the ESM-2 and ProtT5 models to extract the hidden states from their respective final layers. Both pre-trained models are kept frozen and used solely as feature extractors, without fine-tuning on the AIP dataset. Subsequently, mean and max pooling operations applied along the sequence length dimension generate two fixed-size, high-dimensional embedding vectors per model for each peptide. Mean pooling summarizes the overall sequence context, whereas max pooling retains the residue-level signals. Their combination therefore provides complementary representations for short peptides, whose functional patterns may be localized to only a few residues. This end-to-end extraction approach significantly reduces the time required for feature generation.

#### 2.2.2 Supplementary physicochemical and sequence features

Although LLMs effectively capture the implicit semantics of sequences, AIPs are typically short, and the models may struggle to extract structural information from limited local context. Introducing explicit low-dimensional biophysical features mitigates the representational sparsity of language models in the short peptide feature space and stabilizes the decision boundaries of downstream classifiers. Given the distinct data processing mechanisms of tree-based models and deep neural networks, this study extracts global and local multi-scale explicit features tailored to the two respective processing streams within the HD-AIP framework.

1. Global representations for the PLM-fusion machine learning stream
  To accommodate structured-data classifiers, we utilize global composition and distribution characteristics. Specifically, we extract 29-dimensional macroscopic physicochemical features including sequence length, molecular weight, overall hydrophobicity, isoelectric point, aromaticity, instability index, proportion of positively and negatively charged residues (Kawashima *et al.* 2008), and the amino acid composition (AAC) of the 20 standard amino acids. To characterize the spatial amphiphilicity of AIPs, we compute the hydrophobic moment assuming an ideal α-helical conformation based on the Eisenberg hydrophobicity scale (Eisenberg *et al.* 1982). Additionally, we incorporate the composition of k-spaced amino acid pairs (CKSAAP) with the gap parameter set to g∈{0, 1, 2, 3, 4}, which generates an additional 2000-dimensional sequence feature. Combining these properties, the framework successfully maps variable-length peptide sequences into fixed-size, high-dimensional structured vectors.
2. Local representations for CT-Net
  To preserve the spatial topological structure of peptide sequences, the proposed method encodes each sequence into a two-dimensional physicochemical feature matrix with a shape of L × 8. For each amino acid residue, the model first extracts a 7-dimensional basic physicochemical descriptor encompassing hydrophobicity, hydrophilicity, molecular weight, isoelectric point, polarity, aromaticity, and side-chain charge (Kawashima *et al.* 2008). In addition, given the critical role of electrostatic interactions in AIP binding, we compute the global charge density of the entire sequence and append this feature to the 7D descriptor as the eighth feature dimension.

Incorporating these multi-scale explicit features not only enhances the biophysical interpretability of the implicit semantics but also accommodates the specific data formatting requirements of various underlying algorithms.

### 2.3 Heterogeneous dual-stream architecture of HD-AIP

HD-AIP comprises two parallel streams that process peptide representations at different scales, followed by a dynamic ensemble strategy for final prediction.

#### 2.3.1 PLM-fusion machine learning stream

This stream aims to extract discriminative information from high-dimensional global sequence features.

We first concatenate global semantic representations from ProtT5 and ESM-2 with 29 macroscopic physicochemical features and 2000 CKSAAP features. The semantic representations incorporate both mean and max pooling operations, yielding 2048 and 5120 dimensions for the ProtT5 and ESM-2 models, respectively. The total concatenated dimension is 9197.

To prevent the curse of dimensionality and overfitting, we perform tree-based feature selection using a pre-trained LightGBM classifier (Ke *et al.* 2017). Features are ranked according to their importance scores, and the top 300 are retained to train the final LightGBM classifier. The final classifier is configured with 754 boosted trees, a learning rate of 0.0158, 128 leaves, a maximum tree depth of 14, a feature subsampling ratio of 0.5, and a sample subsampling ratio of 0.7734.

#### 2.3.2 Deep parallel network stream CT-Net

To effectively process local topological information, we design CT-Net, a deep parallel network that integrates explicit physicochemical features.

First, the peptide sequence is segmented into 3-mer (k = 3) tokens and mapped to dense semantic embeddings using pre-trained BioVec word vectors (Asgari and Mofrad 2015). Simultaneously, for the residue-level physicochemical matrix of shape L × 8, a multilayer perceptron performs feature dimension expansion and nonlinear enhancement to reduce feature discrepancies across different dimensional scales.

Subsequently, CT-Net feeds these processed features into two asymmetrical branches.

1. Branch 1 (1D-CNN): to capture continuous local motifs under physical constraints, we concatenate the enhanced physicochemical feature matrix and the sequence semantic embeddings along the channel dimension. This concatenated representation is passed through two 1D convolutional modules equipped with residual connections and batch normalization, ultimately extracting local features via global max pooling (He *et al.* 2016).
2. Branch 2 (Transformer Encoder): to capture long-range dependencies among residues, we add positional encodings to the BioVec embeddings and input them to a Transformer encoder with two layers and four attention heads. This module computes global contextual representations via self-attention, followed by mean pooling (Vaswani *et al.* 2017).

Finally, CT-Net concatenates the local motif features from Branch 1 with the global contextual features from Branch 2 and passes the resulting representation through a fully connected layer to produce prediction probabilities. The CNN branch used a kernel size of 3, and dropout rates of 0.2, 0.3, and 0.4 were applied to the embedding layer, the CNN/Transformer branches, and the fully connected layers, respectively. CT-Net was trained for up to 150 epochs with a batch size of 4 using the AdamW optimizer, an initial learning rate of $2\times 10^{-4}$, a weight decay of $1\times 10^{-4}$, and a CosineAnnealingLR scheduler. To improve generalization and mitigate class imbalance, we further employed FGM adversarial training with a perturbation scale of 1.0 (Miyato *et al.* 2017) and focal loss (α=0.45, γ=2.0) to place greater emphasis on challenging samples (Lin *et al.* 2017).

### 2.4 Dynamic ensemble strategy

At the decision level, we evaluated two fusion methods: stacking and dynamic weighted soft ensemble.

Based on the stacking framework (Wolpert 1992), the prediction probabilities of the two streams are concatenated into a two-dimensional vector, utilizing logistic regression as a meta-learner. However, stacking performed poorly on imbalanced data (reduced MCC and accuracy) and was prone to overfitting due to the low-dimensional feature space. Consequently, the HD-AIP framework ultimately adopts a dynamic soft ensemble strategy. For a given peptide, CT-Net and the PLM machine learning stream generate prediction probabilities $P_{CT}$ and $P_{ML}$, respectively. The final prediction probability $P_{\mathrm{final}}$ of the system relies on their weighted sum, formulated as follows:


(1) Pfinal=ω·PCT+(1-ω)·PML,

where the fusion weight ω∈[0, 1] and the classification decision threshold are optimized through grid search during FFCV. Unlike stacking, this method avoids introducing an additional meta-classifier and extra trainable parameters. This not only reduces the risk of overfitting but also allows for adaptive weighting, effectively balancing overall accuracy and minority-class recall.

To ensure the reproducibility of our results, all hyperparameter configurations—including those for the LightGBM classifier, the CT-Net architecture, and the dynamic ensemble strategy—are comprehensively detailed in the Supplementary Material.

### 2.5 Evaluation metrics

The evaluation process utilizes six standard metrics commonly applied in binary classification tasks. These metrics encompass accuracy (ACC), precision, Sn, Sp, MCC, and AUC.

Given the limited overall sample size and the class imbalance, we adopted MCC as the primary comprehensive evaluation metric instead of the traditional F1 score. By incorporating all four dimensions of the confusion matrix—true positives, true negatives, FP, and false negatives—the MCC calculation effectively prevents the model from biasing predictions toward the majority class. This mathematical characteristic reduces evaluation discrepancies introduced by sample imbalance. The specific formulas are defined as follows:


(2) ACC=TP+TNTP+TN+FP+FN,# 

(3) Precision=TPTP+FP,

(4) Sn=TPTP+FN,

(5) Sp=TNTN+FP,

(6) MCC=TP×TN-FP×FN(TP+FP)(TP+FN)(TN+FP)(TN+FN). 

## 3 Results

### 3.1 Multi-source feature ablation in the PLM-fusion stream

To quantify the contribution of each feature domain in the PLM fusion machine learning stream, we conducted a feature-level ablation experiment. All feature sets were reduced to the top 300 dimensions via LightGBM importance ranking to ensure fair comparison and mitigate dimensionality issues. FFCV results are presented in Table 3 and Fig. 3a.

**Figure 3:**
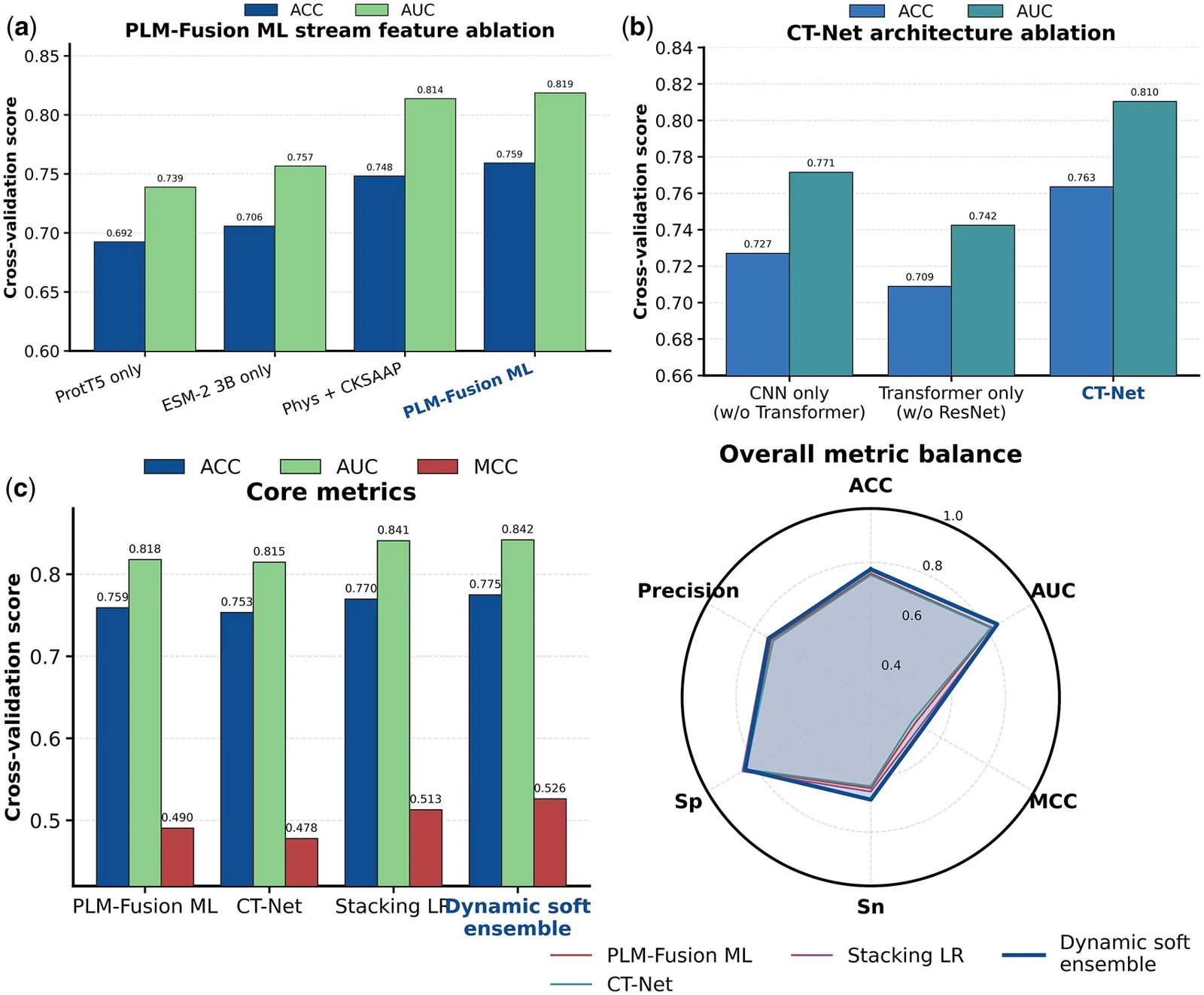
Internal ablation analysis of the HD-AIP framework. (a) Ablation analysis of multi-source features. (b) Ablation analysis of the CT-Net architecture. (c) Performance comparison of single-stream baselines and decision-level ensemble strategies.

**Table 3:** Ablation study of multi-source features in the PLM-fusion ML stream.

Feature Combination	Original dimension	Selected dimension	ACC	AUC
ProtT5 only	2048	Top 300	0.6924	0.7388
ESM-2 3B only	5120	Top 300	0.7058	0.7566
Phys + CKSAAP only	2029	Top 300	0.7482	0.8137
**PLM-fusion ML stream**	**9197**	**Top 300**	**0.7592**	**0.8186**

Note: Bold values indicate the best ACC and AUC results among the compared feature combinations.

Among single-domain features, traditional physicochemical and compositional features (Phys + CKSAAP) achieved the strongest baseline performance (ACC = 0.7482, AUC = 0.8137). In contrast, using ProtT5 or ESM-2 3B alone as frozen extractors yielded lower metrics, indicating that explicit handcrafted features remain critical for AIP sequences, which rely on specific physicochemical constraints such as charge distribution and hydrophobic patterns. Fusing all three domains after consistent feature selection produced the highest overall performance, confirming the complementarity between deep semantic embeddings from LLMs and traditional physicochemical features. The tree-based selection mechanism effectively isolates the most discriminative subset from the high-dimensional heterogeneous space.

### 3.2 Architecture ablation of CT-Net

CT-Net is designed as a dual-branch architecture that simultaneously captures local residue motifs (via a 1D-CNN residual branch) and global long-range dependencies (via a Transformer encoder). We performed architecture-level ablation under FFCV, comparing the complete CT-Net against two single-branch variants: (i) CNN only (removing the Transformer branch) and (ii) Transformer only (removing the CNN branch). Results are summarized in Table 4 and Fig. 3b.

**Table 4:** Internal ablation study of the CT-Net architecture.

Architecture Configuration	ACC	AUC
w/o transformer (CNN only)	0.7270	0.7715
w/o CNN (Transformer only)	0.7089	0.7424
**CT-Net**	**0.7635**	**0.8104**

Note: Bold values indicate the best performance among the compared architecture configurations.

The complete CT-Net outperformed both single-branch variants, confirming the necessity of the dual branch design. Removing the CNN branch (Transformer only) caused a substantial performance drop (AUC from 0.8104 to 0.7424), indicating that for relatively short AIP sequences, the 1D-CNN plays a critical role in capturing essential continuous local motifs; omitting convolution reduces Sn to interactions among adjacent residues. Conversely, removing the Transformer branch (CNN only) also reduced AUC to 0.7715, demonstrating that convolutional layers alone cannot adequately model long-range dependencies between distant residues due to their limited receptive field. These results show that short-range motifs and long-range dependencies are complementary, and their parallel integration effectively mitigates feature bias when processing short peptides.

### 3.3 Decision-level ensemble strategy comparison

After extracting features via the two streams, we compared two decision-level fusion strategies: logistic regression-based stacking and dynamic soft ensemble. Single-stream baselines (PLM-fusion ML stream and CT-Net alone) were also evaluated. Results are shown in Table 5 and Fig. 3c.

**Table 5:** Performance comparison of single-stream baselines and different decision-level ensemble strategies.

Model	ACC	AUC	MCC	Sn	Sp	Precision
PLM-fusion ML stream	0.7592	0.8178	0.4905	0.6377	0.8402	0.7269
CT-Net	0.7532	0.8147	0.4778	0.6305	0.8351	0.7183
Stacking LR	0.7697	0.8409	0.5129	0.6502	**0.8494**	**0.7422**
**Dynamic soft ensemble**	**0.7749**	**0.8419**	**0.5261**	**0.6800**	0.8382	0.7371

Note: Bold values indicate the best performance for each evaluation metric among the single-stream baselines and decision-level ensemble strategies.

Single-stream baselines showed comparable performance, indicating that neither macroscopic tree-based models nor microscopic deep networks alone can fully capture all discriminative information. Stacking LR improved AUC and Sp but yielded only marginal gains in ACC and MCC. In contrast, dynamic soft ensemble achieved the best overall performance, particularly on MCC (0.5261) and Sn (0.6800), demonstrating better adaptability to class imbalance. The optimal weight obtained via grid search was $P_{\mathrm{final}}=0.52\cdot P_{CT}+0.48\cdot P_{ML}$, indicating that CT-Net’s local motif features contribute slightly more (52%) while the global features provide complementary validation (48%).

### 3.4 Comparison with traditional machine learning classifiers

Using the same top 300 fused features (ProtT5, ESM-2 3B, Phys, AAC, CKSAAP), we compared LightGBM against other traditional classifiers [SVM, Random Forest, XGBoost (Chen and Guestrin 2016)] under FFCV. The top 300 features were selected through a unified tree-based feature selection mechanism before classifier comparison. This experiment isolates the effect of the classifier choice while keeping the feature space identical. Results are summarized in Table 6 and Fig. 4a.

**Figure 4:**
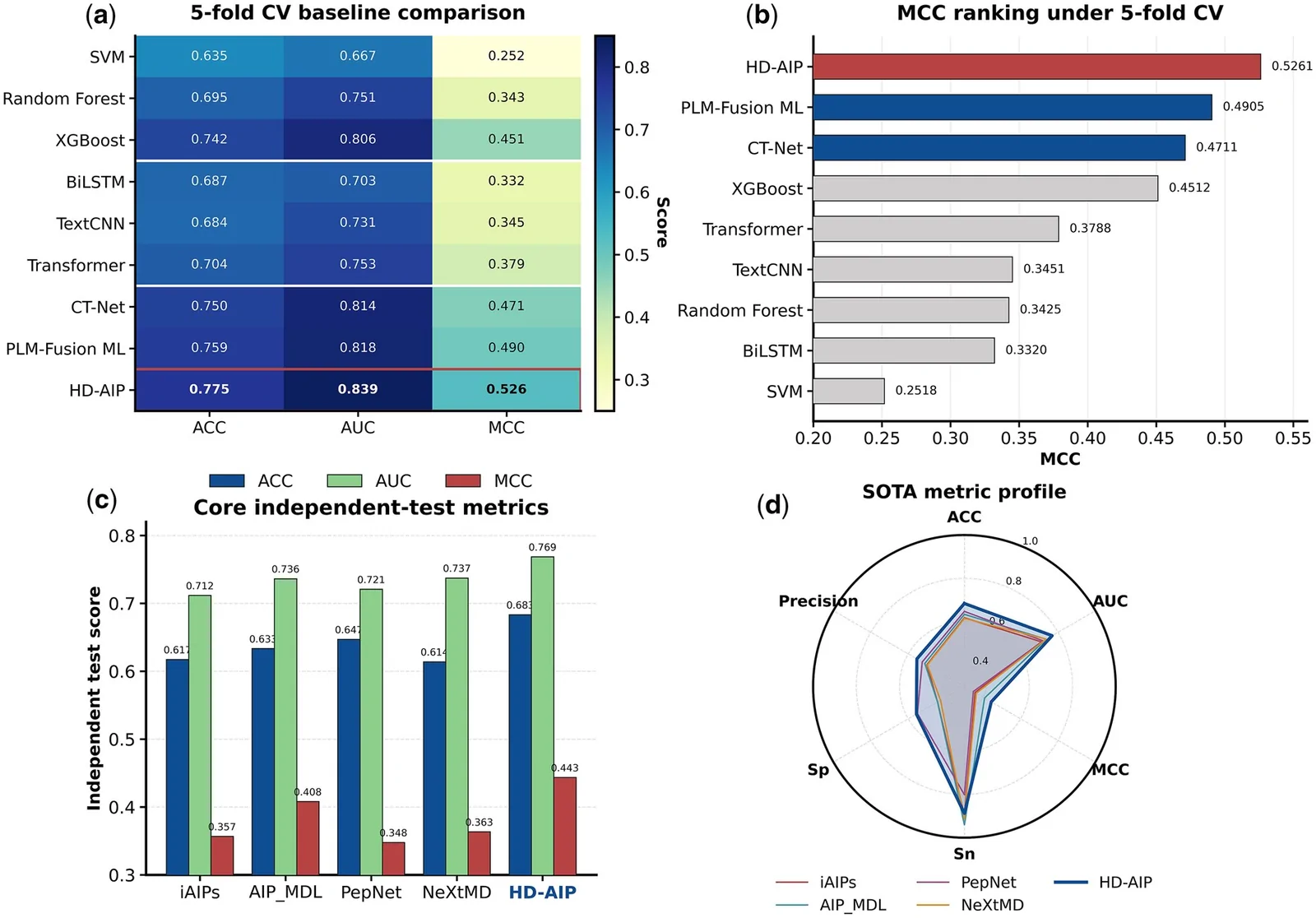
Comprehensive performance evaluation of HD-AIP under cross-validation and independent testing. (a) Heatmap of ACC, AUC, and MCC under FFCV. (b) MCC-based ranking of all evaluated baseline models under FFCV. (c) Comparison of core independent-test metrics. (d) Radar plot showing the overall independent-test metric profile.

**Table 6:** Comprehensive performance comparison between HD-AIP and traditional machine learning classifiers.^a^

Specific model	ACC	AUC	MCC
SVM (linear)	0.6354	0.6666	0.2518
Random forest	0.6950	0.7508	0.3425
XGBoost	0.7420	0.8061	0.4512
PLM-fusion ML (single stream)	0.7592	0.8178	0.4905
**HD-AIP (dynamic ensemble)**	**0.7747**	**0.8394**	**0.5261**

a The HD-AIP results in this table were obtained from an independently repeated run of the same evaluation pipeline and may therefore differ slightly from those in Table 5.

Note: Bold values indicate the best performance for each evaluation metric among the compared methods.

As shown in Table 6, linear SVM performed poorly across all metrics (ACC = 0.6354, MCC = 0.2518), confirming that the deep feature manifold of AIPs remains highly nonlinear even after dimensionality reduction. Random Forest and XGBoost achieved stronger nonlinear fitting, with XGBoost reaching an AUC of 0.8061 and MCC of 0.4512. However, LightGBM consistently outperformed both, achieving the highest ACC (0.7592), AUC (0.8178), and MCC (0.4905). This demonstrates that LightGBM benefits from gradient-based one side sampling and exclusive feature bundling, which improve classification robustness on heterogeneous feature spaces combining continuous hig-dimensional semantics with discrete low-dimensional physical constraints.

### 3.5 Comparison with classic DL architectures

Using the same inputs as CT-Net (BioVec embeddings + 8-dimensional residue-level physicochemical features), we compared CT-Net against three classic DL models: BiLSTM, TextCNN (Kim 2014), and standard transformer (Vaswani *et al.* 2017). All models were evaluated under identical FFCV. Results are shown in Table 7 and Fig. 4b.

**Table 7:** Comprehensive performance comparison between HD-AIP and classic DL architectures.

Specific model	ACC	AUC	MCC
BiLSTM	0.6867	0.7027	0.3320
TextCNN	0.6845	0.7314	0.3451
Standard transformer	0.7043	0.7535	0.3788
CT-Net (single stream)	0.7499	0.8141	0.4711
**HD-AIP (dynamic ensemble)**	**0.7747**	**0.8394**	**0.5261**

Note: Bold values indicate the best performance for each evaluation metric among the compared methods.

BiLSTM struggled to model long-range electrostatic interactions in spatially folded peptides. TextCNN captured local motifs but had a fixed receptive field, limiting its global perspective. The standard Transformer achieved the best performance among classic DL models via self-attention, but directly concatenating physical and semantic features before network processing made it vulnerable to dimensional noise from physical quantities on small datasets, causing semantic overfitting. CT-Net’s advantage stems from its asymmetric routing design: physicochemical constraints (enhanced by an MLP) are injected unidirectionally into the CNN branch to establish explicit local physical boundary constraints, while the Transformer branch operates in a pure semantic space to coordinate global dependencies. Additionally, focal loss and FGM adversarial training contributed to improved generalization on imbalanced boundary samples.

### 3.6 Comparison with state-of-the-art methods

To further evaluate generalization capability in real-world scenarios, we compared HD-AIP with four recent state-of-the-art tools—iAIPs (Zhao *et al.* 2021), AIP_MDL (Guan *et al.* 2023), PepNet (Han *et al.* 2024), and NeXtMD (Xie *et al.* 2025)—on an independent test set (303 sequences, strictly non-overlapping with the training set). These tools represent distinct technical trajectories: iAIPs uses random forest with handcrafted sequence features; AIP_MDL employs a stacking ensemble of extra trees, GRU, and CNN with attention; PepNet utilizes ProtT5 embeddings with residual dilated convolutions and transformers; NeXtMD combines four ML classifiers with a ResNeXt module. Results are presented in Table 8, Fig. 4c and d.

**Table 8:** Performance comparison of HD-AIP with state-of-the-art tools on the independent test set.

Module	ACC	AUC	MCC	Sn	Sp	Precision
iAIPs	0.6172	0.7116	0.3567	0.8966	0.4439	0.5000
AIP_MDL	0.6333	0.7362	0.4079	**0.9396**	0.4438	0.5117
PepNet	0.6469	0.7207	0.3477	0.8017	0.5508	0.5254
NeXtMD	0.6139	0.7373	0.3634	0.9138	0.4278	0.4977
**HD-AIP**	**0.6832**	**0.7686**	**0.4434**	0.8879	**0.5561**	**0.5538**

Note: Bold values indicate the best performance for each evaluation metric among the compared methods.

HD-AIP achieved the highest ACC, AUC, MCC, Sp, and precision on the independent test set. While AIP_MDL and NeXtMD exhibited very high Sn (0.9396 and 0.9138, respectively), their Sp was extremely low (0.4438 and 0.4278), indicating a strong bias toward FP that would hinder practical high-throughput screening. PepNet showed relatively better balance but still underperformed HD-AIP on all metrics except sensitivity. In contrast, HD-AIP maintained a well-balanced profile (Sn = 0.8879, Sp = 0.5561), demonstrating robust generalization and effective false-positive control on unseen data. This advantage stems from: (i) multi-scale feature construction integrating dual language models with physicochemical properties; (ii) the CT-Net asymmetric design that imposes physical constraints only on the CNN branch while preserving a pure semantic space for the Transformer; and (iii) the dynamic soft ensemble that adaptively adjusts decision weights and thresholds without introducing additional trainable parameters.

## 4 Conclusion

In this study, we presented HD-AIP, an alignment-free heterogeneous dual-stream framework for predicting AIPs. By directly constructing multi-scale representations from individual peptide sequences without relying on homology alignment, the framework is well-suited for high-throughput virtual screening. The core contribution lies in the parallel integration of macroscopic global semantics—via dual protein language models (ProtT5 and ESM-2 3B) combined with sequence-level physicochemical features—and microscopic local topological motifs, captured by an asymmetric CT-Net. A dynamic soft ensemble strategy adaptively merges the two streams without introducing additional trainable parameters.

Extensive experiments, including internal ablation studies, comparisons with traditional machine learning and classic DL baselines, and evaluations against state-of-the-art tools, demonstrate that HD-AIP achieves superior and balanced predictive performance. The framework maintains high Sn while substantially reducing false-positive rates—a persistent limitation of many existing methods—and its effectiveness on an independent test set confirms robust generalization to unseen sequences.

Several limitations should be acknowledged. Embedding extraction from LLMs still demands considerable computational resources, and the independent test set, though strictly deduplicated, is relatively modest in size. The current framework also does not incorporate explicit three-dimensional structural information. In addition, the moderate Sp (Sp = 0.5561) indicates room for further improvement to reduce FP during candidate screening and alleviates the burden of downstream experimental validation, thereby enhancing HD-AIP’s utility for screening and prioritizing candidate AIPs.

Future work will explore four directions: lightweight model distillation to improve computational efficiency; integration of predicted three-dimensional conformations to enrich feature representations; optimization of the decision threshold and training strategy to improve specificity, for example through cost-sensitive learning and hard-negative mining; and multi-task learning frameworks to jointly model AIPs alongside other functionally related host defense peptides, such as antimicrobial and immunomodulatory peptides. These extensions aim to further enhance HD-AIP’s applicability in large-scale peptide discovery campaigns.

## Supplementary Material

btag697_Supplementary_Data

## Data Availability

You can find the source code and dataset needed on GitHub (https://github.com/Zerofly0/HD-AIP). The required package files have been listed in the README.
