# Supplementary material for: HD-AIP: a heterogeneous dual-stream alignment-free framework for anti-inflammatory peptide prediction based on language models and CT-Net
Source: Bioinformatics. 2026 Sep 21;42(10):btag697. doi: 10.1093/bioinformatics/btag697 (PMC13637612; doi:10.1093/bioinformatics/btag697)
Supplement: Supplementary file 1 [file btag697_supplementary_data.docx]

Appendix A: Implementation Details and Hyperparameter Configurations

| **Module** | **Hyperparameter** | **Value** | **Description** |
| --- | --- | --- | --- |
| **Global Settings** | Cross-validation Folds | 5 | Number of folds for rigorous evaluation |
|  | Random Seed | 2026 | Ensures experimental reproducibility |
|  | Batch Size | 4 | Number of samples per training batch |
| **PLM-Fusion ML Stream**  *(LightGBM Classifier)* | n_estimators | 754 | Number of boosted trees |
|  | learning_rate | 0.0158 | Shrinkage rate for tree updates |
|  | num_leaves | 128 | Maximum tree leaves for base learners |
|  | max_depth | 14 | Maximum tree depth |
|  | colsample_bytree | 0.5 | Subsample ratio of columns when constructing each tree |
|  | subsample | 0.7734 | Subsample ratio of the training instances |
|  | reg_alpha (L1) | 0.0049 | L1 regularization term on weights |
|  | reg_lambda (L2) | 0.0001 | L2 regularization term on weights |
|  | min_child_samples | 55 | Minimum number of data needed in a child |
| **CT-Net**  *(Deep Learning Stream)* | Epochs | 150 | Maximum training iterations |
|  | Optimizer | AdamW | Optimizer with weight decay regularization |
|  | Base Learning Rate | 2e-4 | Initial learning rate |
|  | Weight Decay | 1e-4 | L2 penalty coefficient |
|  | LR Scheduler | CosineAnnealingLR | Adjusts LR following a cosine curve |
|  | 1D-CNN Kernel Size | 3 | Kernel size for extracting local motifs |
|  | Transformer Layers | 2 | Number of encoder layers |
|  | Attention Heads | 4 | Number of parallel attention heads |
|  | Dropout Rate | 0.2 - 0.4 | Applied to embeddings (0.2), CNN/Transformer (0.3), and FC layers (0.4) |
|  | FGM Perturbation () | 1.0 | Adversarial training perturbation scale |
| **Dynamic Ensemble** | Weight for CT-Net | 0.52 | Optimized via grid search |
|  | Cutoff Threshold | 0.46 | Optimized via grid search |
